# Designer mammalian living materials through genetic engineering

**DOI:** 10.1016/j.bioactmat.2025.02.007

**Published:** 2025-02-15

**Authors:** Mariana Gameiro, José Almeida-Pinto, Beatriz S. Moura, João F. Mano, Vítor M. Gaspar

**Affiliations:** CICECO-Aveiro Institute of Materials, Department of Chemistry, University of Aveiro Campus Universitário de Santiago, Aveiro, 3810-193, Portugal

**Keywords:** Genetic engineering, Synthetic biology, Tissue engineering, Living materials, Mammalian cells

## Abstract

Emerging genome editing and synthetic biology toolboxes can accurately program mammalian cells behavior from the inside-out. Such engineered living units can be perceived as key building blocks for bioengineering mammalian cell-dense materials, with promising features to be used as living therapeutics for tissue engineering or disease modeling applications. Aiming to reach full control over the code that governs cell behavior, inside-out engineering approaches have potential to fully unlock user-defined living materials encoded with tailored cellular functionalities and spatial arrangements. Dwelling on this, herein, we discuss the most recent advances and opportunities unlocked by genetic engineering strategies, and on their use for the assembly of next-generation cell-rich or cell-based materials, with an unprecedent control over cellular arrangements and customizable therapeutic capabilities. We envision that the continuous synergy between inside-out and outside-in cell engineering approaches will potentiate the future development of increasingly sophisticated cell assemblies that may operate with augmented biofunctionalities.

## Highlights

• Mammalian cell units can be genetically engineered with programmable instructions.
• Efforts have been made to merge synthetic biology, materials, and tissue engineering.
• Cell-dense assemblies can possess intricate architectures and enhanced functionality.
• Genetically designed materials may unlock promising biomedical applications.

## Graphical abstract

**Figure:**
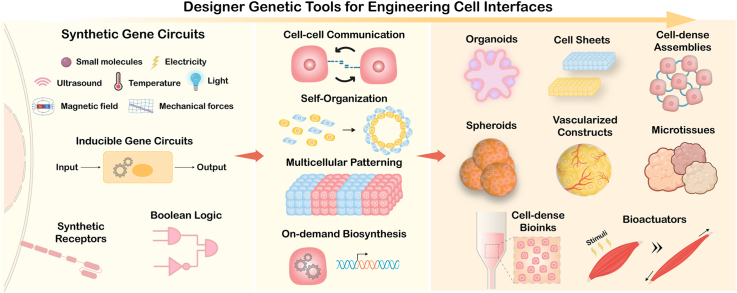

## 1 Introduction

Mammalian living materials are rapidly emerging as sophisticated living therapeutics, as well as platforms for tissue engineering and disease modeling applications [[1], [2], [3]]. Essentially, living materials rely on exploiting the fundamental units of life – cells – as living building blocks to bioengineer multi-scale and cell-rich assemblies, either solely comprised of cells or combined with supporting biomaterials in a minimalistic mode [1,2,4,5]. As opposed to conventional biomaterial-based platforms, living materials present a distinguished cell density, rendering materials with improved degrees of biological complexity, biofunctionality, maturation, and responsiveness [1,5]. Outside-in engineering strategies leveraging materials science conventional top-down approaches, along with emerging bottom-up engineering strategies, have been thoroughly explored to advance hierarchical living architectures fabrication. For instance, these living structures are commonly obtained either by: (i) remodeling the extracellular milieu, (ii) controlling the deposition of micro-tissues, or (iii) engineering cell surfaces, allowing for a certain degree of freedom in the assembly stage of living constructs [2,6]. Such living architectures have been shown to be particularly interesting to address specific medical applications, such as the acceleration of angiogenesis, or tissue-specific repair (e.g., skin, heart, or bone) [1]. Nonetheless, the physicochemical cues of outside-in strategies cannot ensure a high spatiotemporal resolution over cell distribution, fate and functionality, limiting the control of the biological behavior of living assemblies [2,7,8]. Also, smart materials and exogenous growth factors cannot control specific intracellular pathways, hindering an accurate programming of mammalian cells [7].

On the other hand, engineering the cell fate from inside-out, leveraging genetic engineering and synthetic biology toolboxes has emerged as an alternative approach for fine-tunning mammalian living systems manufacture, with a higher degree of control over cell units along time [1,7,8]. From a mechanistic view, living cell units can be perceived as tiny machines, waiting for user-defined or microenvironment induced biocomputing instructions [9]. Programming cellular building blocks with switchable genetic circuits enables high spatiotemporal control over cell behavior with improved predictability [[10], [11], [12], [13], [14], [15]]. Moreover, emerging genome editing technologies can now be used to allow permanent manipulation of mammalian genomes with an unprecedent precision [16,17]. Hence, the use of genetic strategies may be necessary to ensure dynamic biological features within living assemblies, such as cellular autonomy, immune modulation, complex cell-cell communication pathways, and inducible behaviors [7,8].

By synergistically combining both frameworks (outside-in and inside-out), we may expect a new era of mammalian living materials that can be assembled and programmed on-demand with tunable behavior and functionality.

We make use of the term “machine” to emphasize their architectural complexity integrated with the inherent mechanistic genetic programmability for driving cells to perform functions that are user-encoded [7,8,18,19]. In this review, we focus on the promise behind genetic-based engineering strategies for advancing such next-generation living materials from inside-out. Notable efforts to orchestrate the self-organization and biofunctionality of multicellular mammalian systems, namely organoids [20,21], synthetic tissues [22,23], biobots [24,25], as well as smart therapeutic artificial bioimplants [26,27] are here showcased. Looking ahead, we propose that genetically engineered mammalian living materials can be engineered with increasing biological complexity, multifunctionality, and adaptability. Ultimately, these may open new frontiers in bioengineering and personalized healthcare applications.

## 2 Genetically engineered cell living units

**Fig. 1:**
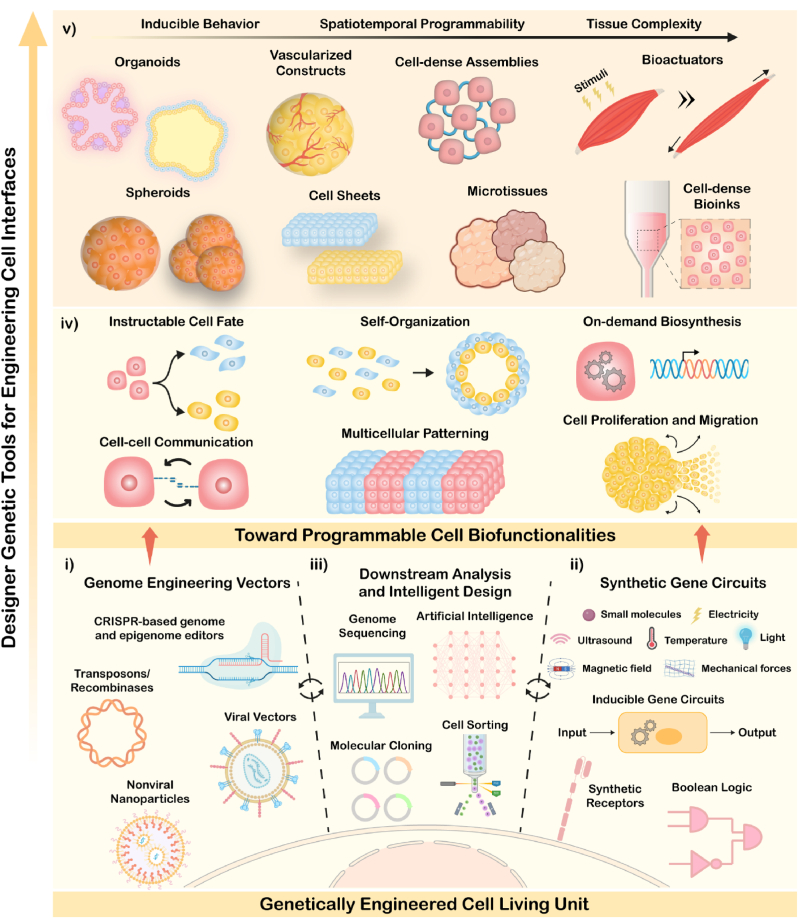
Illustration of genetically programming mammalian cell living building blocks for bioengineering complex living systems. (i) Inside-out cell engineering strategies include major transposon and viral vectors, as well as site-specific CRISPR-based genome and epigenome editors, in which proficient delivery systems are key for efficiently delivering such apparatus into the cells. (ii) Inducible synthetic biology-inspired gene circuits are key for controlling cell behavior. This can be attained by exploiting designed synthetic receptors, closed-loop circuits responsive to exogenous stimuli (e.g., light, small molecules, sound), and open-loop feedback circuits responsive to specific physiological cues. Logic gated circuits are also key for achieving cellular biocomputing programmability. (iii) In parallel, downstream analysis and intelligent design approaches aid in the efficient and rapid development of such engineering toolboxes, harnessing emergent artificial intelligence algorithms, next-generation genome sequencing, cell sorting strategies, and molecular cloning. (iv) At a cellular level, different living behaviors can be meticulously controlled, including on-demand synthesis of specific biomolecules, cell fate and differentiation, proliferation and migration, as well as cell-cell communication and gene expression patterning. (v) For fabricating truly living mammalian systems, inside-out engineering can be interfaced with outside-in engineering approaches (e.g., materials science, biofabrication and tissue engineering), for developing complex designer constructs with high biofunctionality and spatiotemporal programmability. Organoids, spheroids, vascularized constructs, cell sheets, programmable cell-dense assemblies and microtissues, dynamic bioactuators and bioinks can be engineered for human therapeutics, regenerative medicine, disease modelling, and soft robotics.

Currently, outside-in strategies have been particularly valuable to generate living structures that found applicability in multiple bioengineering fields (i.e., disease modeling, drug testing, tissue repair, etc.). Advances in bottom-up strategies and bioengineering tools (i.e., metabolic glycoengineering, covalent conjugation, etc.) have enabled an initial control over the self-assembly process of cellular units into living architectures. In addition to this, mammalian cell living units can be meticulously rewired from inside-out by leveraging a wide range of genetic engineering toolboxes (Fig. 1) [11,17]. Leveraging on this vast toolbox that is currently available, one can envision that cells may be genetically engineered and manipulated not only during the assembly process but also over time, allowing an enhanced spatiotemporal control over cell organization and behavior of generated living architectures. Despite the extensive research on transient delivery of gene-encoded plasmids or mRNA for reprogramming cell units behavior and bioactivity, the permanent and stable integration of whole transgene cassettes into the genome may also contribute for creating a new generation of cell and gene therapies [16,28]. Stable integration of transgenes is of particular interest in *ex vivo* cell engineering, and may be generally attained with viral vectors (e.g., retro- or lentiviruses) or via transposons (e.g., Sleeping Beauty or PiggyBac) in a fully or semi-random manner [8,28].

On the other hand, site-specific gene editing tools have rapidly emerged in recent years, evolving from early Cre recombinases, zinc finger nucleases (ZFNs) and transcription activator-like effector nucleases (TALENs), to today's renowned CRISPR-based editors [16,17,[29], [30], [31]]. These precise tools can virtually target any genomic loci, enabling site-specific knock-in/out, and integration of transgenes within known genomic safe harbors (e.g., AAVS1, or CCR5) [28,32]. Moreover, cutting-edge base and prime editors enable more reliant and safer gene editing applications while presenting minimum toxicity in cells [30,33,34]. Beyond this, CRISPR-based toolboxes can be leveraged for epigenome engineering, essentially by modulating gene transcription without altering the genome [17,35]. In the field, different toolsets have been developed to date, namely CRISPRa/i, or CRISPRon/off platforms, exploiting deactivated nucleases, transcriptional activation or repression domains, and chromatin modifiers [17,29,35].

The use of synthetic biology approaches for programming mammalian cells has received increasing attention in the last decade of tissue engineering, especially for the implementation of customized gene circuits to instruct cell behavior [11,12,36,37]. Designer cells can for example sense a user-defined input, process it and respond to it [12,37]. Open-loop gene switches entail a user-defined responsiveness to endogenous or traceless physical cues (e.g., small molecules, light, heat, or sound, etc.), while closed-loop feedback devices enable on-demand responses to physiological cues, such as disease-related biomarkers [15,[37], [38], [39], [40]]. Additionally, synthetic biologists have so far also designed numerous cell-surface synthetic receptors for triggering custom signaling cascades, such as CARs [41], synNotch [42], Tango [43], MESA [44], Cha-Cha [45], or GEMS [46,47].

Envisioning advanced cell biocomputing, layered Boolean logic gated circuits can also be used to promote fine-control over stimuli-responsive cells, following a series of logic operations (i.e., AND, OR, NOR, or NAND gates), as largely explored in emerging CAR-T cell engineering [11,12,36,48]. The advancement of the above-mentioned genetic devices often require iterative design-build-test cycles, harnessing different molecular cloning techniques (e.g., Gibson assembly, Gateway cloning, etc.) combined with fluorescence-activated cell sorting (FACS), PCR and DNA sequencing steps [7,12,49]. Up-to-date, this major focus on genetic cell engineering has also entailed the optimization of the delivery such genetic machinery into mammalian cells [50,51]. Massive efforts have been put into tailoring physical methods, such as electroporation, as well as viral and nonviral systems (e.g., peptides, lipids, or polymers) [15,50,51]. Such emerging genetic engineering and synthetic biology toolboxes have thus considerably advanced cell reprogramming applications in recent years.

Drawing inspiration from such breakthroughs it becomes clear that a new bioengineering wave of genetically engineering mammalian living materials with augmented biofunctionalities and intricate architectures has emerged (Fig. 1). Such efforts spark breakthroughs and foster collaborative efforts from tissue engineers and synthetic biologists, as it will be showcased in the following sections.

## 3 Advancing genetically tailored mammalian living materials

Genetically engineered mammalian living materials have recently emerged for a wide range of biomedical applications elevating tissue engineered platforms toward complex biological constructs with defined programs. By exploring such tools, cells can be programmed from the inside-out to tune their behavior along time, allowing researchers for example to: (i) shape architecture and functionality of assembled living structures and/or (ii) encode smart features in living therapeutics, as will be showcased in the following subchapters.

### 3.1 Shaping the architecture and functionality of multicellular assemblies

**Fig. 2:**
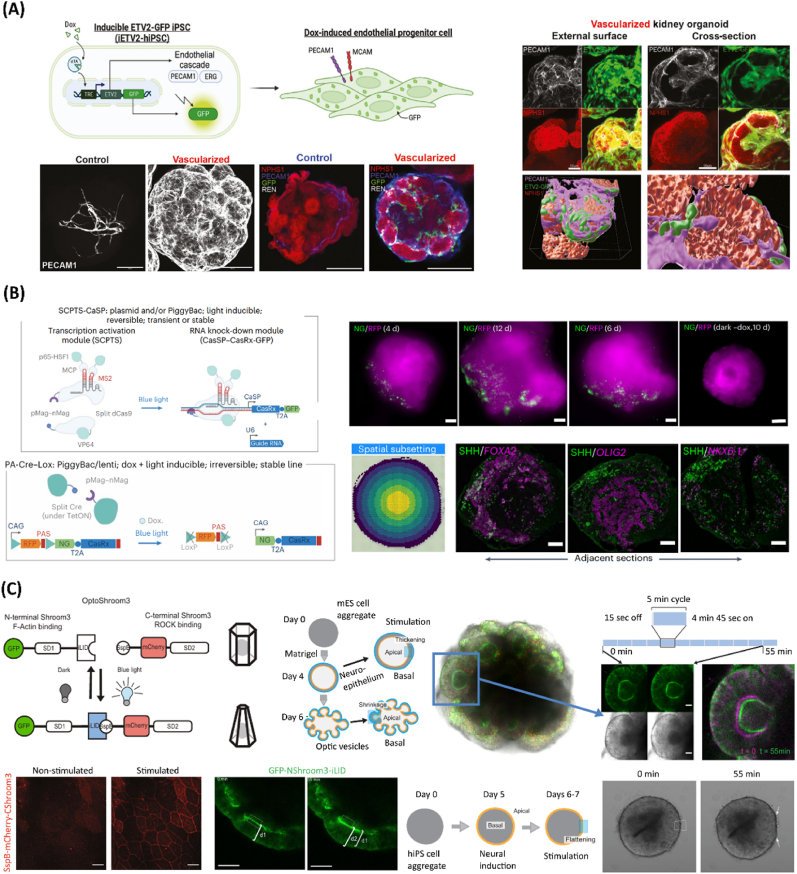
Strategies and tools for genetically guiding the self-organization and maturation of next-generation organoids. A) Inducible, vascularized human kidney organoid. Top left: Schematic of PiggyBac-engineered dox-inducible ETV2-human induced pluripotent stem cell line (iETV2-hiPSC). Bottom Left: Representative immunofluorescent platelet and endothelial cell adhesion molecule 1 (PECAM-1) images of endothelial cell network in vascularized and control kidney organoids; Images showing emergence of a renin (REN) cell population in vascularized kidney organoid, and no REN^+^ cells in control. Right: Vascularized kidney organoids with GFP^+^ endothelial cells encasing the podocyte clusters from the external surface. Reproduced with permission [55]. Copyright 2024, Elsevier. B) Optogenetic patterning of a human neural organoid. Top left: Schematics of light-inducible transcription activation module (SCPTS), based on a dCas9 fused to transcription activation domains, driving CasRx transcription; A U6 promoter-driven CasRx guide RNA can be co-expressed. Bottom left: Schematics of PiggyBac-engineered dox and light-inducible Cre-Lox system, based on a split Cre fused with pMag-nMag photodimers. Top right: Spatial photostimulations by a LED array, in which images represent an organoid (4–12 days), locally photostimulated via laser scanning. Bottom right: Optogenetic stimulation of SHH in neural organoids, coupled with spatial readouts, in which images represent SHH-expressing cells in adjacent cryo-sections of organoids with laser induction of SHH in the north-west pole. Reproduced with permission [21]. Copyright 2023, Springer Nature. C) Optogenetic control of apical constriction in a human neural organoid for induced tissue deformation. Top left: OptoShroom3 genetic constructs, based on dimerization of Shroom Domain 1 (SD1) and Shroom Domain 2 (SD2) upon blue light illumination; Schematics of formation and stages of mouse optic vesicle organoids regarding OptoShroom3-induced tissue deformation. Bottom left: Images of Madin-Darby Canine Kidney (MDCK) cells expressing SspB-mCherry-CShroom3 before and after 1 min stimulation; Images of organoid thickness before and after stimulation. Top right: Optic vesicle organoid and stimulation cycles, altering the lumen diameter of optic vesicles. Bottom right: Images of OptoShroom3-induced flattening through local stimulation of neuroectodermal organoids. Reproduced with permission [56]. Copyright 2022, Springer Nature.

Diving into synthetic tissue development and morphogenesis, organoids can be perceived as 3D self-organizing entities. These can generally be derived either from human embryonic stem cells (hESCs), induced pluripotent stem cells (hiPSC) or from adult stem cells (hASCs) [31]. Such biometic microtissues can truly recapitulate human tissue early developmental states or functionalities, serving also as unique *in vitro* disease modeling platforms [13,18,52,53]. To surpass bioengineering challenges and essentially build increasingly biomimetic organoids, various genetically-guided organoids have emerged in recent years (Fig. 2). Early attempts have leveraged dox-inducible expression of the transcription factor GATA6 for assembling complex fetal liver organoids with recapitulative human bud- and vascular-like phenotypes [54].

In an elegant approach, informative computational transcriptomics analyses have been explored to enable the rational design of matured structures with superior hepatic features and vascularity by the overexpression of key transcription factors (i.e., ATF5, PROX1) and CRISPR-based activation of a specific enzyme (i.e., Cytochrome P450 3A4 (CYP3A4)) [20]. Notably, vascular structures play a key role in the organogenesis, fate maturation and patterning of organoids [13,57]. By inducing the expression of the transcription factor ETV2, researchers were also able to genetically guide the establishment of vascular networks within hESCs-derived brain [57], and hiPSCs-derived kidney organoids [55]. In the latter, the generation of extensive endothelialization was key to drive a native-like multilineage kidney maturation with a humanized cellular pool, with the resulting organoids harboring matured podocytes, interstitial cells, and renin cells (Fig. 2A) [55]. Synthetic organizer-like signaling centers can also be explored to program the self-organization of organoids [13]. By chemically inducing a polarized activation of long-range signaling Wnt/β-catenin in embryoid bodies (EBs) [58], or Sonic Hedgehog (SHH) pathways in brain organoids [59], patterned topographies could be attained, mimicking *in vivo* early tissue development. Alternatively, optogenetic circuits have been exploited for spatiotemporally orchestrating SHH-patterned and polarized brain organoids with biomimetic human neurodevelopment traits (Fig. 2B) [21]. By combining light-inducible genetic modules with single-cell and spatial transcriptomics, researchers could characterize the patterning modalities in such constructs [21]. The ability to precisely direct such 3D tissue deformation as described before can be in turn useful for shaping intricate architectures in mammalian systems. As a proof-of-concept, researchers have discovered a way to reversibly fold neural organoids with optogenetics, leveraging Shroom3, a key regulator of apical constriction of morphogenetic processes in vertebrates [56]. For instance, multiple 3D tissue deformations, including thickening, flattening and lumen shrinkage could be achieved, recapitulating mammalian 3D dynamic morphogenesis events (Fig. 2C) [56]. Finally, cutting-edge gene editing toolboxes can be exploited for generating advanced isogenic *in vitro* human disease models, by accurately recapitulating specific genome mutations or gene variants related to certain diseases [31,[60], [61], [62]]. For instance, proficient and versatile protocols have been developed for electroporated base and prime editing-based generation of isogenic ASC hepatocyte, endometrium, and colon organoid lines [60,61].

**Fig. 3:**
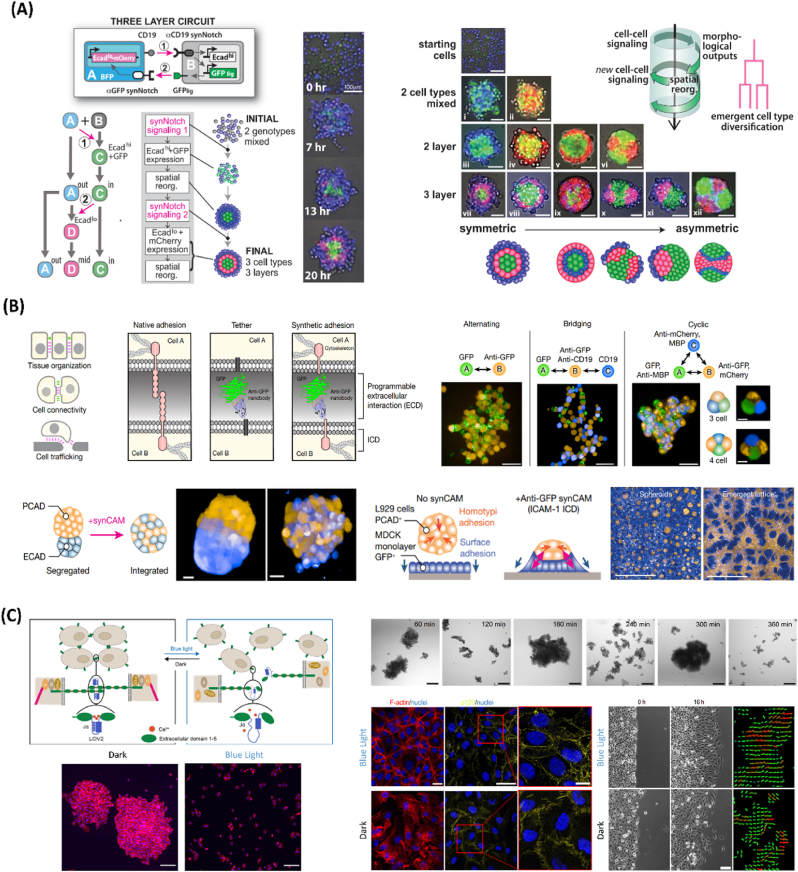
Bottom-up genetic programming of synthetic multicellular assemblies. A) synNotch-based programming of cell assemblies. Left: Schematics of three-layer circuit, in which an A-type cell sends signals to a B-type cell using CD19 ligand, inducing high expression of E-cadherin and GFP; Induced B-type cell then sends reciprocal signals to A-type cell, and GFP serves as ligand to stimulate anti-GFP synNotch receptor expressed in A-type cell; In the bottom, cell fate diagram showing how the process evolves, self-organizing into three distinct cell phenotypes organized into three spatially distinct compartments; Images of spheroids development with a three-layer architecture, from 0 to 20 h. Right: Representative schematics of different self-organizing multicellular structures programmed via synNotch toolsets. Reproduced with permission [66]. Copyright 2018, American Association for the Advancement of Science (AAAS). B) synCAM-based multicellular assembly and tissue remodeling. Top left: Schematics of functional roles of cell adhesion and design of synCAM receptors, in which the extracellular domain of a CAM is replaced by GFP and a GFP-binding nanobody (anti-GFP). Top right: Custom heterotypic assemblies with alternating, bridging, and cyclic patterning, with L929 cells expressing synCAMs. Bottom left: Exploitation of synCAMs technology to force the integration of differentially sorting L29 populations, leading into a binodal structure. Bottom right: L929 cells mixed with an epithelial MDCK monolayer, initially forming spheroids on top of the monolayer, and later converging into a complex lattice-like network, after the introduction of GFP-anti-GFP synCAMs, thus reshaping tissue organization. Reproduced with permission [22]. Copyright 2022, Springer Nature. C) Opto-E-cadherin-based reversible programming of cell-cell adhesions. Top left: Schematics of Opto-E-cadherin platform, in which cells that express opto-E-cadherin on their surfaces form cell-cell adhesions in the dark but not with blue light; LOV2 domain is inserted between the first and second extracellular domains E-cadherin in proximity to one of the calcium binding sites. In the dark, the Jα-helix of the LOV2 remains folded such that the Ca2+ ions can bind and the E-cadherins on neighboring cells interact. Under blue light, the Jα-helix unfolds such that the Ca2+ ions cannot bind and the E-cadherin interactions are lost. Bottom left: Images showing Opto-E-cadherin-MDA cells after 4 h in the dark (aggregated) or under blue light. Top left: Brightfield images of opto-E-cadherin-MDA cells in suspension culture under repeated 60 min dark/blue light cycles and their clustering dynamics, showing temporal and bidirectional control over cell-cell adhesions. Bottom middle: Fluorescence images showing light-controlled actin cytoskeleton reorganization in the dark or under blue light in 2D cultures, with F-actin (red), nuclei (blue), and p120 (yellow) staining. Bottom right: Bright field images of opto-E-cad-MDA cells in a wound healing assay in the dark and under blue light for 16 h. Reproduced with permission [67]. Copyright 2022, Springer Nature.

Besides stem-cell derived self-organized systems, artificial genetic programs can be exploited for building preprogrammed synthetic multicellular assemblies from the bottom-up (Fig. 3) [13,52,53,63]. Inspired by the proposed Turing patterns found in embryonic development (e.g., zebra skin stripes), reaction-diffusion mathematical models can be explored for shaping mammalian patterns (i.e., inducing spots, or stripes, etc.) [52,63]. By manipulating the Nodal-Lefty signaling pathway – key in embryonic patterning – activator-inhibitor gene circuits can be reconstituted for inducing positive feedback of short-range Nodal activator and negative feedback of long-range Lefty inhibitor [64]. Moreover, cell adhesion molecules, such as cadherins, can be strategically manipulated for guiding cell adhesion-based patterns [13,63]. For instance, chemogenetic circuits for cadherin-based cell sorting systems can induce 2D and 3D striped patterns in cell aggregates bearing differentially expressed cadherin types [65]. More recently, a synNotch receptor system, based on the core regulatory domain of the juxtacrine signaling receptor Notch, connected to a chimeric extracellular recognition domain, and a chimeric intracellular transcriptional domain, has been explored for advancing the generation of synthetic, mammalian-origin symetric>asymetric tissue-like architecutres (Fig. 3A) [13,42,46,63]. In essence, upon recognizing a specific ligand on a neighboring cell, the transmembrane region of the receptor undergoes cleavage, thus releasing the intracellular transcriptional domain to enter the nucleus and drive the expression of user-defined genes [66]. Therefore, synNotch enables the generation of customized genetic programs to control cadherin expression via cell-cell communication and synthetic signaling cascades, enabling the bioengineering of multi-layered patterned mouse fibroblast-derived spheroids (Fig. 3A) [66]. Through cyclic logic sequences of cell signaling interactions and induced morphological rearrangements, iterative refinements of multicellular self-organization can be accomplished.

The reproducible and customizable features of synNotch demonstrate its vast potential for bottom-up tissue engineering, having been later reengineered for detection of soluble synthetic morphogens [68]. In another approach, and aiming towards increasing complex architectures, the helixCAM platform has been designed for programming patterning formation [69]. Based on the surface presentation of coiled-coil peptides, fused to a transmembrane domain, this system enables the fabrication of different multicellular aggregates in human leukemia cells, including five-layered spherical assemblies, and allows the manipulation of cell's migration and morphology [69]. Additionally, the Lim Lab has also recently generated novel synthetic cell adhesion molecules (synCAMs) by combining orthogonal extracellular binding domains with native intracellular domains (Fig. 3B) [22]. In this strategy, synCAMs can be used to dictate cellular morphology and cytoskeletal structure along with customizable cell-cell patterns [22]. Moreover, such tools can be exploited for tissue remodeling by enabling: (i) the integration of different cell populations into a singular spherically-structured multicellular structure, and (ii) by enabling the self-remodeling and convergence between fibroblasts spheroids and epithelial monolayers into a complex lattice-like tissue network (Fig. 3B) [22]. In the future, such genetically programmed structures could expedite sophisticated platforms for tissue engineering and repair. Additionally, genetic circuits can be leveraged for precisely programming differential cellular states within a single cell type [22,[70], [71], [72]]. Leveraging zinc finger transcription factors, the MultiFate platform enables to irreversibly guide up to seven different cellular states, regulated by external small molecules [70]. Moreover, a stochastic recombinase genetic switch has been explored for inducing downstream fates with tunable subpopulation ratios and 3D morphologies, from a monoclonal population [71]. Finally, different optogenetic circuits have emerged for assembling cellular building blocks into tissues [13,73,74]. For instance, the opto-E-cadherin platform enables the spatiotemporal remote programming of E-cadherin-mediated cell-cell adhesions and organization of the actin cytoskeleton, directing multicellular aggregation and migration (Fig. 3C) [67]. Interestingly, repeated ON-and-OFF (dark/blue light) switching cycles could be used to promote dynamic and reversible manipulation of cell assemblies [67]. Such technologies may expedite research toward bottom-up multicellular assemblies with intricate levels of complexity, rendering more dynamic and mimetic mammalian living systems.

**Fig. 4:**
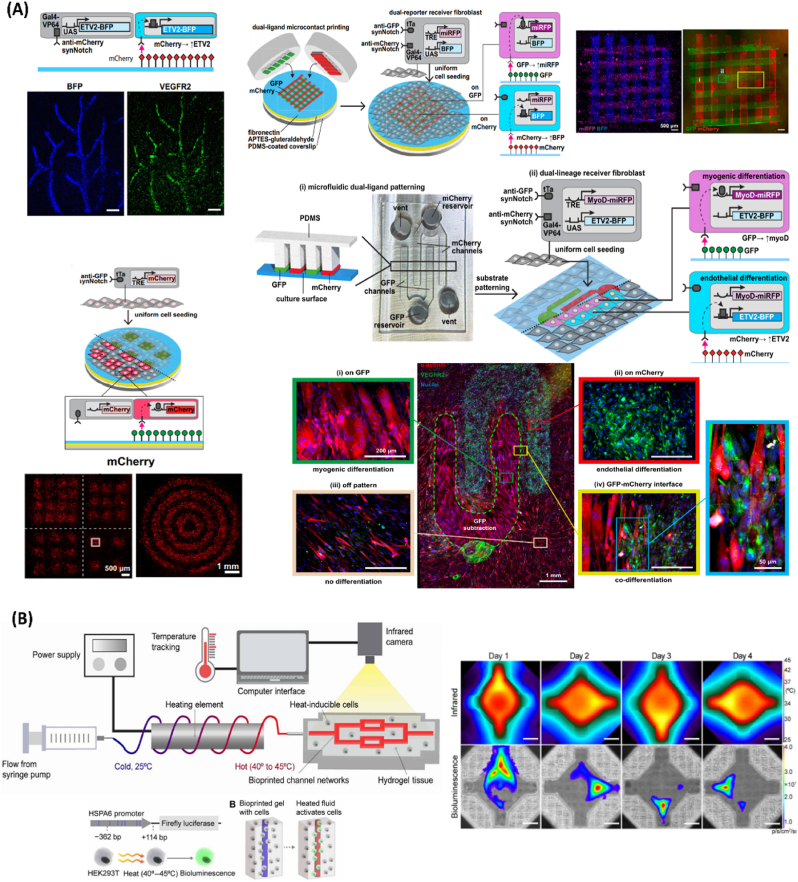
Upscaling genetically programmed and patterned living cell assemblies. A) Material-to-cell synNotch-based programming of multicellular constructs. Top left: Schematics of receiver fibroblasts with anti-mCherry/Gal4 synNotch that activates BFP and ETV2 when cultured on substrates with mCherry, promoting endothelial differentiation; Vasculature-like pattern with resulting fluorescence microscopy images after three days. Top right: Schematics of dual-ligand microcontact printing and seeding of dual-receiver fibroblasts with anti-GFP/tTa synNotch, activating miRFP and anti-mCherry/Gal4-VP64 synNotch that activates BFP; Fluorescence images of microcontact-printed perpendicular lines of GFP an mCherry and corresponding miRFP and BFP expression by dual-receiver cells, after one day. Bottom left: Schematics of anti-GFP/tTA synNotch receiver fibroblasts that activate mCherry cultured on substrates microcontact-printed with GFP; mCherry fluorescence images of patterns, expressed by receiver cells cultured on GFP-patterned substrates, after two days. Bottom right: Schematics of microfluidic patterning platform with alternating rows of GFP and mCherry and corresponding microfluidic device, for promoting spatial co-differentiation of dual-lineage receiver cells into myogenic and endothelial lineages; Dual-lineage receiver fibroblasts with anti-GFP/tTA synNotch that activates MyoD and miRFP and orthogonal anti-mCherry/Gal4-VP64 synNotch that activates ETV2 and BFP, cultured on corresponding substrates patterned with GFP and mCherry; In the bottom, image of dual-lineage receiver cells cultured on the patterned substrates, after three days. Reproduced with permission [75]. Copyright 2024, Springer Nature. B) Heat-inducible regulation of gene expression in artificial tissues. Top left: Schematics of HEAT (heat exchangers for actuation of transcription) platform, in which a biocompatible fluid flows around a power supplied heating element to preheat the fluid before entry in perfusable channel networks within hydrogel tissue constructs laden with heat-sensitive cells. During heating, the hydrogel temperature is continuously monitored using an infrared camera. Bottom left: Schematics of HEK293T cells engineered to express luciferase (fLuc) under HSPA6 promoter. Right: Representative infrared and bioluminescence expression images of dynamic hydrogel activation in different days, showing different activated gene expression patterns through space and time. Reproduced with permission [78]. Copyright 2020, American Association for the Advancement of Science (AAAS).

A convergence between synthetic biology with materials science can indeed drive the assembly of increasing scalable living constructs (Fig. 4) [8,19]. For instance, the synNotch technology has been recently upgraded into a highly versatile material-to-cell programmable tool for spatially controlling tissue organization with user-defined gene expression patterns and cell fates [75,76]. Different biomaterials, such as cell-produced extracellular matrix proteins and hydrogels, can be engineered with presenting synthetic ligands (e.g., GFP and mCherry) (Fig. 4A) [75]. These offer a powerful approach for locally controlling genetic networks within customizable patterned mammalian living materials [75]. Also, microfluidic substrates dually patterned with GFP and mCherry ligands have enabled a selective and simultaneous co-transdifferentiation of dual-lineage receiver fibroblasts into myogenic and endothelial lineages [75]. Such dual differentiation could be induced within a continuous tissue construct with custom tissue pattern geometries and alignments, without the need of the addition of soluble differentiation factors [75]. In the future, such technology could be combined with cell-dense bioinks for building 3D bioprinted macro-scale patterned constructs or even for self-orchestrating organoids maturation [75]. Markedly, 3D bioprinting has already began to be integrated with designer cells for achieving intricate control of living architectures. In a recent study, bioinks composed of dox-inducible-transcription-factor hiPSCs were harnessed for assembling multicellular neural tissue-like architecture [23]. This approach enabled the user-defined patterned co-differentiation of neural stem cells, endothelium and neurons in an intricate layered architecture, mimicking the native tissue [23]. From an upscale perspective, holding remote and spatiotemporal genetic control over bioprinted assemblies could be particularly interesting. In this line, centimeter-scale, cell-dense synthetic microgels have been recently developed by combining embedded extrusion-volumetric printing with optogenetic-engineered cells [77].

Such small-scaled tissues featured light-triggered insulin-producing pancreatic beta-like cells in designated patterns, showcasing highly functional microgels [77]. Moreover, by loading heat-inducible cells in bioprinted hydrogels with perfusable channel networks, researchers engineered HEAT platforms – heat exchangers for actuation of transcription (Fig. 4B) [78]. In essence, these can be exploited for achieving depth- and scale-flexible control over volumetric heat-inducible gene patterning in 3D printed artificial tissues, with both spatial and temporal tunability [78]. Such strategy may potentially be interesting for future applications in tissue engineering and regenerative medicine. In other approaches, genetic programs have been further exploited to assemble machine-like living materials with actuation, life-like motions or even unnatural features, potentially advancing a new era in soft biohybrid robotics with user-programmed functionalities [18,24,79,80]. Over the last years, multiple optogenetic circuits have been designed for controlling muscle tissue contraction within robust synthetic constructs [79,[81], [82], [83], [84], [85]]. Such circuits could for instance remotely program multidirectional walking capabilities in skeletal biobots [85], along with flagellar dynamic swimming movements within functional neuromuscular units [79]. Such advancements illustrate the promise behind programmed living materials for achieving increasingly intelligent living systems.

Moving forward, the translation of novel cell-cell signaling programmable toolboxes in pluripotent cell lines is envisioned to potentially enable truly refined *de novo* organoid platforms with customized patterning and cell fate decisions [13,86]. In this focus, Fussenegger's Lab seminal works could serve as a prime ground for the future, with numerous developments in cell therapies with advanced genetic circuits, from disease feedback closed-loop, to music- and mind-controlled gene tuning [14,39,[87], [88], [89]].

### 3.2 Encoding smarter living bioimplants for precision therapy

To fully unlock the implementation of genetic engineering tools in the design of living materials, cells may be encoded with smarter features to improve their therapeutic potential [1,3]. Followed by the wide success of current cell-based therapies, genetically-programmed living assemblies made of mammalian cells may achieve unprecedent therapeutic performances for next-generation personalized interventions. Notably, over the last years, a multitude of synthetic gene circuits have been built for ensuring increasingly robust cell-based therapeutics targeting various human conditions, from cancer, to autoimmune, or metabolic disorders [12,14,16,38].

**Fig. 5:**
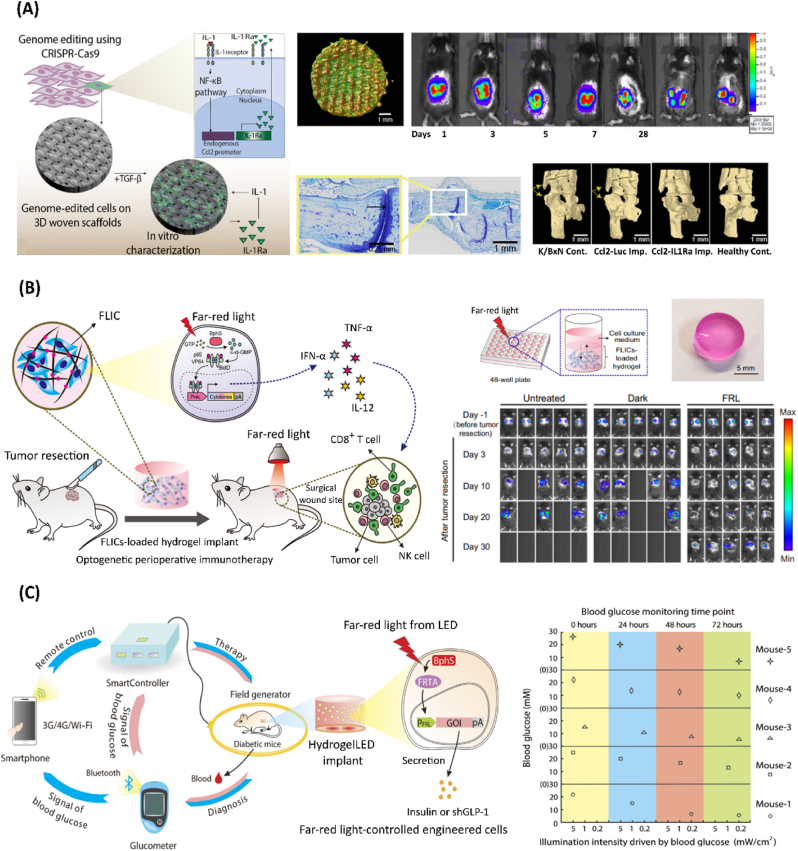
Bioengineering smarter therapeutic mammalian living materials. A) Gene edited self-regulated anti-cytokine bioimplant for treatment of inflammatory diseases. Left: Schematics of CRISPR/Cas9-based iPSCs engineering with a synthetic gene circuit expressing IL-1Ra, in response to activation of the Ccl2 promoter. Cells were loaded on 3D cartilaginous woven constructs in chondrogenic media. Top center: Gene edited cells on porous 3D woven scaffold (nano-computed tomography). Top right: Longevity of implanted constructs, demonstrated by consistent luciferase expression, in mice. Bottom center: Implants reduced inflammation. Bottom right: Mice treated with Ccl2/IL-1Ra implants demonstrate reduced bone damage. Reproduced with permission [26]. Copyright 2022, American Association for the Advancement of Science (AAAS). B) Optogenetic immunotherapeutic construct for cancer treatment. Left: Schematics of optogenetic perioperative immunotherapy mediated by far-red light-controlled immunomodulatory engineered cells (FLICs), encapsulated within a polysaccharide-based hydrogel. Top right: Mixture of far-red light-controlled immunomodulatory designer cells (FLICs) with hydrogel polysaccharide solution, promoting crosslinking of hydrogel matrix, through crosslinking by ions interactions from the cell culture medium and solidified. Bottom right: *In vivo* bioluminescence imaging of tumor recurrence, being significantly reduced only under far-red light (FRL) illumination, with an LED array (λ=730 nm, 1 mW/cm^2^). Reproduced with permission [27]. Copyright 2022, Springer Nature. C) Smartphone-controlled optogenetic implant for treatment of diabetes. Left: Schematics of smartphone-optogenetically regulated electronic system. Smartphone-remote controlled field generator of HydrogelLED implants induce expression of insulin of shGLP-1. Signals of blood glucose are sent via bluetooth to electronic system for autonomously activating the therapeutic system. Right: LED intensity activating designer cells, as reported on the smartphone. Reproduced with permission [102]. Copyright 2017, American Association for the Advancement of Science (AAAS).

Inspired by such advancements, early attempts have focused on genetically upgrading cell sheets [90], spheroids [91], or scaffold-supported assemblies [[92], [93], [94], [95], [96]]. For instance, genetically augmented tissue regeneration or wound healing interventions have been realized, by exploiting the overexpression of key transcription factors, or knock-in of therapeutic gene cassettes within multicellular assemblies [92,94]. Nonetheless, the incorporation of synthetic biology-inspired designer cells within biologically relevant tissue constructs is envisioned to expedite therapeutic living systems (Fig. 5). The Guilak lab has achieved various progresses regarding preprogrammed smart bioimplants that can autonomously respond to biologic cues, in a machine-like dynamic behavior [26,[97], [98], [99], [100]]. For instance, hydrogel-based bioimplants have been combined with chondrocytes harboring mechanogenetic circuits, leveraging the mechanosensory channel TRPV4 found in cartilage tissues [100]. Upon physiological mechanical loading, such designer constructs could sense and self-regulate the release of IL-1 receptor antagonist (IL-1Ra) on-demand, protecting tissues from pro-inflammatory conditions [100]. In another approach, CRISPR/Cas9-edited iPSCs have been harnessed for bioengineering living cartilage tissue and for assembling self-regulatory anti-cytokine therapies, within 3D woven scaffolds [26] (Fig. 5A) and agarose rod-shaped cartilaginous implants [101]. Essentially, by sensing the inflammatory IL-1, such biological systems could respond, in a feedback-manner, with physiological levels of IL-1Ra, leveraging the inducible macrophage chemoattractant protein-1 (Ccl2) promoter [26,101].

In the future, such closed-loop artificial implants could be explored in different human disease conditions towards autonomous and robust cell-based drug delivery platforms. Moreover, far-red light-controlled immunomodulatory designer cells (FLICs) have been loaded into hydrogels for achieving remote and traceless control over cancer-targeted immunotherapeutic interventions (Fig. 5B) [27]. Upon on-demand illumination, such bioimplant could release INF-β, TNF-β, and IL-12 cytokines, promoting long-term activity against tumor recurrence after surgery, and ultimately extending animal survival rate [27]. Bringing sophisticated electrical and software principles into mammalian living systems could further add key layers of complexity and functionality. As a proof-of-concept, smartphone-regulated optogenetic hydrogel constructs have been developed for diabetes treatment (Fig. 5C) [102]. In such platform, user-controlled smartphones could remotely activate light-emitting diodes within the hydrogel, which in turn could induce the release of a short variant of human glucagon-like peptide 1 (shGLP-1) or insulin from optogenetically-engineered cells [102]. In the same system, bluetooth-activated glucometers could further be harnessed for real-time measurement of physiological *in vivo* glycemic levels, automatically triggering a technological cascade in a wireless closed-loop network fashion [102]. Such multidisciplinary designs are highly promising for expediting personalized and translational living drugs. Futuristic research leveraging smart-watch [103] and electrogenetic scaffolds [87,104] for programmable therapeutic transgene regulatory systems are envisioned to advance the next-generation of therapeutic mammalian living materials. Additionally, novel genetically targeted chemical assembly (GTCA) technologies have recently leveraged genetically engineered neurons for *in situ* assembly of polymers within robust living neural networks, opening horizons for next-generation biological interfaces for brain wearable devices and beyond [105,106].

Overall,we before described some of the major examples of genetically engineered mammalian living materials and applications, along with the inside-out strategies and cell types employed in their fabrication, and summarize them in the following Table 1.

**Table 1:** Major examples of genetically engineered mammalian living materials and applications.

Inside-Out Strategy	Engineered Cell Unit	Application	Year	Ref.
Optogenetic activation of SHH, Cre-Lox system	hiPSCs	Neurodevelopmental patterning in brain organoid	2023	[21]
Optogenetic activation of OptoShroom3, PiggyPac	hiPSCs,	Reversible 3D deformations in neural organoid	2022	[56]
Dox-inducible activation of *ETV2*, Tet-On system	hESCs	Functional vascularized and matured brain organoid	2019	[57]
Base Editors for multiplexed mutations	Hepatocyte organoids	Modeling of colorectal tumorigenesis in organoid	2023	[60]
Dox-inducible upregulation of transcription factors, PiggyBac	hiPSCs	Differentiation and vascularization in organoids and bioprinted tissues	2022	[23]
SynNotch signaling, Lentivirus	L929 murine fibroblasts	Spatial self-organization of multicellular structures	2018	[66]
Optogenetic control of E-cadherin	MDA-MB-231, HeLA, L929	Reversible modulation of multicellular adhesions	2023	[67]
MultiFate circuit with zinc finger transcription factors	CHO-K1	Multiple stable and heritable states within multicellular assemblies	2022	[70]
Material-to-cell synNotch signaling, lentivirus	L929 mouse fibroblasts	Spatially controlled cell differentiation in multicellular constrcts	2024	[75]
Optogenetic activation of insulin secretion, Sleeping Beauty	1.1E7 cell line	Spatial patterning of cell differentiation in 3D printed microgels	2023	[77]
Optogenetic activation of neural stimulation	Mouse embryonic stem cells	Neuromuscular actuation in biohybrid swimmer robot	2019	[79]
Optogenetic-induced neuronal depolarization, TALENs	hiPSCs	Human 3D tissue model of neuromuscular junction	2021	[81]
Il-1α-induced feedback secretion of IL-1Ra, CRISPR	Murine iPSCs	Autoregulated anticytokine tissue engineered therapeutic implant	2021	[26]
Optogenetic-induced release of cytokines, Sleeping Beauty	hMSC-TERT	Immunomodulatory hydrogel implant for cancer therapy	2022	[27]
Dox-induced BMP-2 expression, Lentivirus	Murine mesenchymal stem cells	3D bioprinted tissue for treatment of infectious bone defect	2021	[96]
Mechanogenetic-induced IL-1Ra release, Lentivirus	Isolated primary porcine chondrocytes	Bioartificial cartilage tissue for anti-inflammatory therapy	2021	[100]
Optogenetic-induced insulin release, Sleeping Beauty	Isolated primary porcine chondrocytes	Hydrogel-based implant for smartphone-controlled diabetes therapy	2017	[102]

**Abbreviations:** BMP2: bone morphogenetic protein-2, CRISPR: clustered regularly interspaced short palindromic repeats, Dox: doxycycline, ETV2: human ETS variant 2, hESCs: human embryonic stem cells, hiPSCs: human-induced pluripotent stem cells, hMSC-TERT: telomerase-immortalized human mesenchymal stem cells, IL-1Ra: interleukin-1 receptor antagonist, IL-1Ra: interleukin 1-α, OptoShroom3: optogenetic split-version of Shroom3, synNotch: synthetic notch receptor system, TALENs: transcription activator-like effector nucleases, Tet-On system: tetracycline-controlled Tet-On gene expression system.

## 4 Outlook and future perspectives

The design of mammalian living materials with genetically encodable features has witnessed rapid and promising advancements. Such inside-out technologies have been wisely explored to render engineered tissues with natively found, and user-controllable living features (i.e., vascularization, patterning, adaptability, actuation, etc.), overall improving their capabilities to be explored for disease modeling, tissue engineering and soft robotics, among other applications.

Genome and epigenome editors are rapidly evolving, and emerging platforms for scarless large transgene insertion, such as TwinPE [107], PASTE [108], PASSIGE [109], or STRAIGHT-IN [110], it is envisioed that these could enable the proficient integration of increasingly elaborate synthetic gene cassettes in mammalian cells [17].

Moreover, designer “chassis” cells with more sophisticated switchable behaviors, novel synthetic receptors and signaling pathways are envisioned to expedite the generation of tailorable living architectures [12,36,40,46,111]. Electrogenetics could enable researchers to engineer next-generation bioelectronic living interfaces or even whole synthetic living tissues with electrical tunability [87,112]. Going further, CAR-T cell advancements could also inspire future designs, for instance by leveraging Boolean circuits for assembling logic gated intelligent living systems, or safety switches (e.g., suicide, or ON/OFF operations) for attaining highly controllable cell behaviors [41,48,113]. We anticipate that future advancements in such an early-stage field will be marked by continuous endeavors in inside-out strategies.

Moving forward, structural materials possessing material-genetic interface switches are highly interesting for spatially mediating cell fate or activity of material-embedded designer cells [75,76,114,115]. In this scenario, we envison that future advances related to precision chemistry, microfluidics, and biofabrication are poised and will be key to advance cell-dense living materials [19,52,116]. Gene edited 3D biomimetic organ-on-a-chip technologies could, for example, streamline more clinically valuable humanized isogenic disease models [7]. Also, the combination of designer cells and organ-on-chips containing biosensors could potentially enable real-time monitoring of biological data in the foreseable future.

Innovative synergies between inside-out and outside-in engineering perspectives could unlock next-generation artificial systems encompassing on-demand spatiotemporal control of gene responses and actuation behaviors, programmable cell fate and arrangement, and dynamic remodeling of the extracellular matrix. For instance, the integration of gene edited and closed/open-loop designer cells within mammalian living materials with biologically relevant cell densities (i.e., ‘Cellgels’) [5] could lead to autonomous living systems with enhanced capabilities. The installation of quorum sensing-like systems into these cell-dense platforms could enable self-control of cell populations and stimulate evolution/responsiveness overtime, guiding future development of adaptive/evolving therapeutics [117]. Beyond that, mammalian assemblies with unprecedented cell densities could be of particular interest for minimizing transgene silencing effects, which is a current major hurdle [118,119]. Moreover, engineered living materials harnessing yeast cells or bacteria within bulk materials could inspire creative mammalian living biofactories with self-growing and dynamic features [[120], [121], [122]].

Despite such promising horizons, many challenges can be foreseen given the complex nature of such systems. Similarly to current regulatory hurdles for clinical translation found by engineered tissues and cell-based products, genetically enabled living materials are expected to encounter significant obstacles related to the efficiency of gene delivery vehicles, as well as the manufacturing, reproducibility, cell source standardization, and safety [7,16]. Moreover, ethical implications may also rise as synthetic biology may empower researchers for encoding novel/non-natural features [13,18]. iPSCs-derived therapeutic assemblies could vastly gain from specific gene edits for reducing potential traces of immunogenicity [16,123]. Also, technologies such as single-cell CRISPR-based screenings can be highly advantageous, enabling high-throughput analysis of gene regulatory pathways *in vitro* in organoids [31]. In the future, a potential challenge may be to ensure the functionality of cells within living materials over extended periods, which may hinder their final application. Emerging strategies such as synthetic gene oscillators [124], or dCas9-based epigenetic editors [125] may be interesting to explore for slowing cellular aging and maximizing the longevity of engineered materials. Artificial intelligence (AI) and machine learning (ML) models, along with omics technologies will also be key for accelerating cell engineering designs with predictable outcomes [12,15,17,126]. Particularly, emerging deep learning, AI and ML approaches are envisioned to fast-track more in-depth programming over mammalian multicellular systems, by designing, predicting, and optimizing synthetic circuits performances, as well as exploring new artificial gene circuitry and vector design avenues [15,127,128]. Altogether, the advancement of genetically programmable mammalian living materials has a significant potential to contribute for tackling major challenges in biology and biomedicine.

## CRediT authorship contribution statement

**Mariana Gameiro:** Writing – original draft, Methodology, Investigation, Data curation, Conceptualization. **José Almeida-Pinto:** Writing – review & editing, Visualization, Methodology, Data curation. **Beatriz S. Moura:** Writing – original draft, Formal analysis. **João F. Mano:** Writing – review & editing, Supervision, Resources, Funding acquisition. **Vítor M. Gaspar:** Writing – review & editing, Validation, Supervision, Resources, Project administration, Conceptualization.

## Ethics approval and consent to participate

Ethics approval and consent to participate are not applicable to this review.

## Data availability

No data was analyzed or created in this review.

## Declaration of Competing Interests

The authors declare that they have no conflict of interest.
